# Periodic and Aperiodic EEG Features as Potential Markers of Developmental Dyslexia

**DOI:** 10.3390/biomedicines11061607

**Published:** 2023-06-01

**Authors:** Chiara Turri, Giuseppe Di Dona, Alessia Santoni, Denisa Adina Zamfira, Laura Franchin, David Melcher, Luca Ronconi

**Affiliations:** 1 School of Psychology, Vita-Salute San Raffaele University, 20132 Milan, Italy; 2 Division of Neuroscience, IRCCS San Raffaele Scientific Institute, 20132 Milan, Italy; 3 Department of Psychology and Cognitive Science, University of Trento, 38068 Rovereto, Italy; 4 Psychology Program, Division of Science, New York University Abu Dhabi, Abu Dhabi 129188, United Arab Emirates; 5 Center for Brain and Health, NYUAD Research Institute, New York University Abu Dhabi, Abu Dhabi 129188, United Arab Emirates

**Keywords:** developmental dyslexia, reading, reading disorder, EEG, signal-to-noise ratio, neurodevelopmental disorder, specific learning disorder, beta rhythm, alpha rhythm, cortical excitability

## Abstract

Developmental Dyslexia (DD) is a neurobiological condition affecting the ability to read fluently and/or accurately. Analyzing resting-state electroencephalographic (EEG) activity in DD may provide a deeper characterization of the underlying pathophysiology and possible biomarkers. So far, studies investigating resting-state activity in DD provided limited evidence and did not consider the aperiodic component of the power spectrum. In the present study, adults with (*n* = 26) and without DD (*n* = 31) underwent a reading skills assessment and resting-state EEG to investigate potential alterations in aperiodic activity, their impact on the periodic counterpart and reading performance. In parieto-occipital channels, DD participants showed a significantly different aperiodic activity as indexed by a flatter and lower power spectrum. These aperiodic measures were significantly related to text reading time, suggesting a link with individual differences in reading difficulties. In the beta band, the DD group showed significantly decreased aperiodic-adjusted power compared to typical readers, which was significantly correlated to word reading accuracy. Overall, here we provide evidence showing alterations of the endogenous aperiodic activity in DD participants consistently with the increased neural noise hypothesis. In addition, we confirm alterations of endogenous beta rhythms, which are discussed in terms of their potential link with magnocellular-dorsal stream deficit.

## 1. Introduction

Among Specific Learning Disorders, Developmental Dyslexia (DD) represents a complex neurobiological condition affecting the ability to read fluently and/or accurately despite adequate education and normal intelligence. Since DD is persistent throughout development and adulthood, early identification and intervention are essential to avoid negative functional repercussions on academic results and psychological well-being. The diagnostic process is currently based on the evaluation of reading skills through standardized neuropsychological and reading tests of different complexity, usually including words, pseudowords and text reading. Furthermore, major sensory and neurological deficits that could explain reading difficulties must be excluded. However, the variability in the manifestation of learning disorders across the lifespan and between linguistic contexts adds complexity to DD assessment [1]. The diagnostic process could be enhanced by introducing neurophysiological measures which might provide more sensible information about cognitive functioning and DD pathophysiology [2,3]. EEG is an optimal method to reach this goal, considering its established capability of providing reliable markers of several other neurological and neurodevelopmental disorders [4] in a rapid and non-invasive way. Moreover, endogenous electrophysiological activity has been shown to explain individual variability in behavioral performance in different cognitive tasks in neurotypical individuals [5,6,7], thus representing a promising candidate to characterize reading processes in DD at the individual level. 

Many studies have described significant alterations of oscillatory EEG activity in children with DD compared to controls when performing different reading-related tasks. A recent review illustrated how these alterations generally regard theta (~4–7 Hz), alpha (~8–12 Hz), and beta (~13–30 Hz) frequency bands [8]. More specifically, children with reading impairments showed increased theta power during pseudowords encoding [9] and phonological processing [10]. Furthermore, compared to controls, children with DD exhibited altered theta lateralization during a phonological task interpreted as the dysfunctional recruitment of linguistic processing areas [11]. Similarly, beta band activity differed in children with reading difficulties at frontal and parieto–occipital sites during reading and linguistic tasks [10,11,12]. Finally, studies reported a failure in reducing alpha power (as compared to resting state) during verbal and visual tasks [10] and a significant alpha power increase at frontal sites in children with DD, which was linked to effortful semantic processing [12]. 

In addition to task-related electrophysiological alterations, spectral analysis revealed anomalies also in the endogenous (i.e., resting-state) neural activity of the DD population. Results from previous studies described increased delta/theta activity and decreased alpha power in children with DD at rest [8,13]. Furthermore, another study showed an asymmetrically distributed alpha and beta power compared to controls [13]. With respect to the relation between endogenous brain activity and reading skills in DD, direct correlations between frontal theta power and spelling abilities [14], but also between parieto-temporal alpha amplitude and pseudowords reading speed [15], were reported. However, both in children and adults with DD, other studies did not find any difference in the amplitude of resting-state oscillations as compared to controls [10,16,17,18]. Additionally, Individual Alpha Frequency (IAF), corresponding to the peak frequency in the occipital alpha activity [19], might represent another important predictor of individual differences in cognitive abilities in DD. In fact, studies on neurotypical individuals reported higher IAF in high vs. poor memory performers [20,21] and in precocious reading children [22], suggesting that endogenous IAF reflects individual differences in cognitive performance [23,24]. Importantly for clinical populations, IAF was also previously associated with cognitive functioning in Schizophrenia Spectrum Disorder [25] and Autism Spectrum Disorder (ASD) [26,27]. 

Overall, considering the picture emerging from these studies, the available evidence on endogenous neural activity in DD appears limited and sometimes conflicting. A possible source of variability may be attributable to the fact that previous studies did not consider how, in the EEG (or MEG) signal, oscillations conflate with aperiodic activity. This component is characterized by a 1/f-like distribution where power exponentially decreases as the frequency increases [28,29] and can be modeled by two essential parameters: the offset and the exponent. The offset represents broadband power across all frequencies and is operationalized as the power level at the lowest frequency, while the exponent represents the strength of the power decay over frequencies and is operationalized as the slope of the power spectrum [28]. When studying neural oscillations, ignoring the influence of aperiodic activity on the periodic one might lead to misinterpretations of power and/or frequency modulations which could be induced by offset/exponent changes [28,30]. In this regard, recent work has underlined the necessity to jointly analyze the periodic and aperiodic components of the signal to avoid such confounds and to investigate their specific contribution to perception and cognition [28,30,31]. 

Aperiodic activity is thought to have a specific functional significance, despite being traditionally considered as noise in EEG recordings [32]. From a neurophysiological perspective, modulations of aperiodic activity are thought to index alterations of the balance between excitatory and inhibitory neuronal activity (i.e., the “E/I” ratio) [33,34]. In particular, a higher exponent (i.e., steeper spectrum) would reflect the predominance of inhibition over excitation, while the reverse applies to a flatter one. Increments of excitation have been linked to a reduction in the coupling between oscillatory cycles and neuronal spiking, determining higher levels of neural noise which may disturb neural communication [35]. With respect to the offset, upward shifts (i.e., higher broadband power) have been linked to increased neuronal firing rates [36], which may partly contribute to the amount of neuronal spikes uncoupled to neural oscillations, thus inducing additional noise.

The aperiodic component dynamically varies within and between subjects as a function of several factors: significant changes in its pattern have been observed during the first year of life [37], through development and early adulthood [38,39,40], and during aging [41,42]. At the intra-individual level, anesthetics-induced changes in subjects’ consciousness levels influence the decaying pattern of the aperiodic power across frequencies [33,43]. Furthermore, both resting-state and task-related aperiodic activity emerged as an important predictor of cognitive indices across several domains (e.g., working memory, short-term memory, processing speed, auditory processing) [28,31,33,42,44]. Moreover, Demuru and Fraschini [45] showed that the aperiodic component allows the accurate identification of individual differences in cognitive processing. Lastly, a recent review underlined the potential role of the aperiodic measures as biomarkers and their usefulness in advancing novel hypotheses about the pathophysiological mechanisms of different psychiatric and neurological conditions [4]. In particular, alterations of the aperiodic activity were found for Attention Deficit Hyperactivity Disorder (ADHD) [46], ASD [47] and Schizophrenia [48], possibly representing an additional neural marker with respect to the oscillatory ones. Notably, several core pathophysiological mechanisms of these neurodevelopmental and psychiatric conditions can be traced back to an imbalanced E/I ratio. This point is particularly relevant for DD considering that, as formalized by the neural noise hypothesis, the evidence suggests alterations of glutamatergic signaling and neuronal migration within important hubs for reading processes, which in turn might induce increased neuronal excitability [49]. Crucially, this theoretical framework could be tested by analyzing aperiodic features of the EEG and their relationship with reading performance at the individual level. 

As reviewed above, analyzing endogenous neural activity in individuals with DD might improve the diagnostic process by providing possible neurophysiological markers. Considering the conflicting evidence in the literature linking anomalies in oscillatory activity to DD and its core deficits, it is essential to elucidate the differential contributions of both periodic and aperiodic activity of the EEG. Indeed, aperiodic features variations could have introduced a certain degree of variability in the previous findings. Moreover, in light of the neural noise hypothesis of DD [49], studying alterations of the E/I ratio as a proxy for neural noise might provide relevant theoretical advancements for understanding its core pathophysiology. Accordingly, the main aim of this study was to deepen the characterization of the endogenous neural activity of individuals with DD by means of a separate analysis of its periodic and aperiodic components while also exploring their possible links to reading abilities. 

## 2. Materials and Methods

We recorded EEG activity at rest in adults with DD and in a group of typical readers. This was completed both with closed and open eyes, as these two states might introduce differential modulations in periodic and aperiodic activity. Relatedly, in some studies comparing individuals with and without DD, resting-state EEG was acquired only with closed eyes, while in others, it was acquired only with open eyes or in both ways [8]. In neurotypical individuals, it is common to observe larger power for theta, alpha and beta frequency bands during eyes-closed (EC) with respect to eyes-open (EO) resting-state EEG [50,51,52]. 

Theta, alpha and beta power—as well as IAF—were extracted both from the raw (uncorrected) and the aperiodic-adjusted (corrected) EEG power spectrum to test potential differences between participants with and without DD. Moreover, we analyzed group differences in the offset and the exponent from the aperiodic activity and investigated whether differential modulations of both periodic and aperiodic activity could predict behavioral performance at several reading tests which are commonly used during the diagnostic process of DD.

### 2.1. Participants

A total of 57 adult participants were included in the study (mean age: 22.2 years; range: 18–30 years). All of them were Italian native speakers, had normal or corrected-to-normal vision, and no history of psychiatric symptoms. In the DD group, 26 participants (17 M; 9 F) had a diagnosis of Developmental Dyslexia certified by a clinical psychologist, while in the Control group, 31 age-matched participants (15 M; 16 F) did not report any reading difficulties in their academic history. The data of 1 participant with DD were excluded from statistical analyses as the EEG recording was corrupted. The data of additional 2 participants with and 3 participants without DD were marked as outliers during the analyses of EEG data (see Section 2.4). The final sample included 51 participants (mean age: 22.3; range: 18–30): 23 DD participants (14 M; 9 F) and 28 Control participants (14 M; 14 F). After giving informed consent, participants underwent a cognitive assessment by means of a short form of the Raven’s Advanced Progressive Matrices (APM) [53]. The results of the assessment are summarized in Table 1. The research project was approved by the Ethical Committee of the University of Trento.

### 2.2. Reading Tasks

Reading skills were assessed using three tasks with different complexity as in our previous study in DD adults [54]. First, participants were administered a text reading task [55] specifically designed to evaluate reading deficits in adult individuals, being highly complex from a linguistic point of view. The other two tasks consisted of reading lists of words and pseudowords extracted from an Italian battery for the evaluation of DD [56], which measure reading skills based on lexical access and grapheme–phoneme-conversion processes, respectively. For the text reading task, performance was assessed in terms of reading time (seconds per syllable), while for the remaining reading tasks, performance was assessed in terms of reading speed (syllables per second). Accuracy was assessed in terms of the number of errors for all tasks. The results of the assessment are summarized in Table 1.

### 2.3. EEG Data Analyses

EEG data were recorded during a resting-state period with an eego^TM^sports cap 64 channels system (ANT Neuro, Enschede, The Netherlands). Electrodes were placed according to the extended international 10–20 system using AFz as the ground electrode and CPz as the online reference. Additionally, an electrooculogram was acquired on the left eye suborbital ridge to monitor eye movements. The online sampling rate was set at 500 Hz, and electrode impedances were kept below 20 kΩ. Sitting on an adjustable chair, participants were instructed to relax and stay as still as possible for 4 min in total. Resting-state EEG was acquired for 2 min with their eyes open (EO) and then closed (EC) for 2 min more.

The acquired data were preprocessed using Matlab version R2020a (MathWorks, Natick, MA, USA) and the EEGLAB toolbox (Swartz Center for Computational Neuroscience, La Jolla, CA, USA) [57]. After being re-referenced to the average across all channels, high-pass (0.5 Hz), low-pass (80 Hz) and notch (50 Hz) filters were applied. The continuous recordings were epoched in 1 s long segments. Spherical interpolation was applied to bad channels, leading to the interpolation of an average of 0.70 (SD = 1.09) bad channels in the EC condition and of 0.78 (SD = 1.18) bad channels in the EO condition. Artifactual components were rejected after being identified by an Independent Component Analysis (ICA). The remaining artifacts were removed by visual inspection, resulting in an average of 2.8% (SD = 0.03) rejected epochs for the EC condition and 2.28% (SD = 0.03) for the EO condition. The final number of artifacts-free epochs per participant included in the analysis was 121.86 (SD = 7.88) for EC and 119.14 (SD = 8.73) for the EO condition. The FFT power spectrum between 1 and 40 Hz was computed using a Hanning taper zero-padded to a length of 4s via FieldTrip toolbox version 20230118 (Radboud University, Nijmegen, the Netherlands) [58] to increase the frequency resolution. The aperiodic (1/f) and periodic (i.e., oscillatory) activity of the power spectrum were modeled via the “Fitting Oscillations and One-Over-f” (FOOOF) [28] plugin in FieldTrip. The offset and exponent parameters were extracted from the models of aperiodic activity for each channel; the first refers to the broadband shift of power across frequencies, while the second refers to the negative slope of the power spectrum (i.e., the “speed” of the power decay across frequencies) [28]. From now on, we will refer to the spectral model of periodic activity as the “periodic power spectrum” and to the spectral model of aperiodic activity as the “aperiodic power spectrum”. Next, we extracted mean power values within the theta (4–6 Hz), alpha (7–13 Hz) and beta (15–25 Hz) bands both from the “raw” power spectrum and from the “FOOOF-corrected” periodic one (hereafter, abbreviated as “FOOOF-corr.”). Individual alpha frequency (IAF) was computed as the frequency with higher power within the alpha band (7–13 Hz) from the “raw” power spectrum and from the “FOOOF-corrected” periodic one.

### 2.4. Statistical Analyses

**Figure 1:**
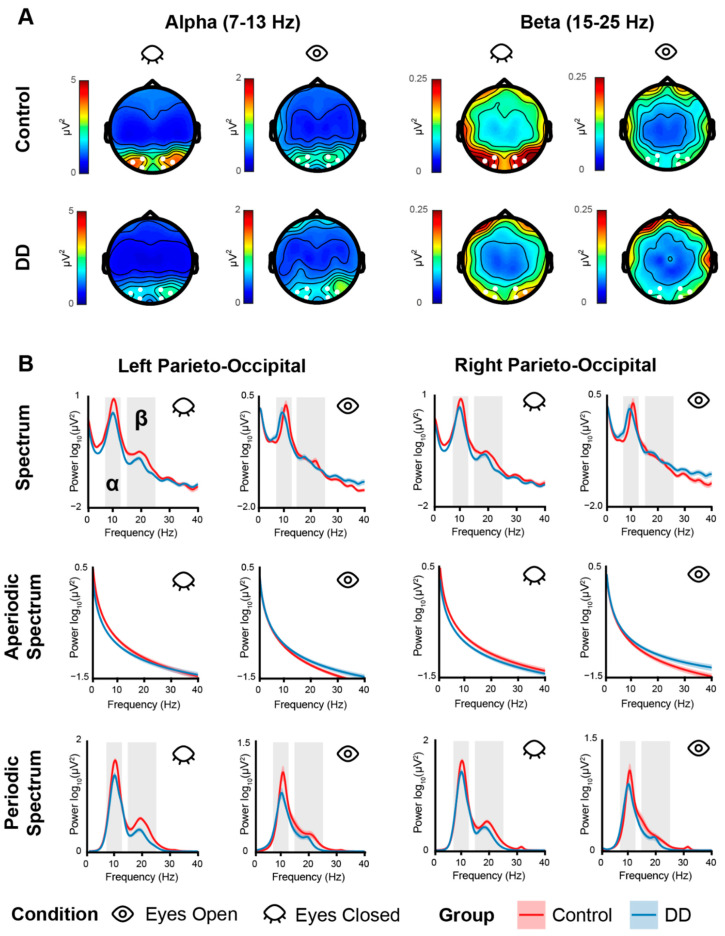
(**A**) Topographies of alpha (1st–2nd columns) and beta (3rd–4th columns) power calculated for each condition (Eyes Closed: odd columns; Eyes Open: even columns) and each group (Control: 1st row; DD: 2nd row). White dots indicate the channels used to compute alpha and beta power for statistical analyses. (**B**) Power spectra. Top row shows the power spectra computed for each group (Control: red line; DD: blue line), each condition (Eyes Closed: odd columns, Eyes Open: even columns), and each electrode cluster (Left Parieto-Occipital: left columns, Right Parieto-Occipital: right columns). The 2nd and 3rd rows show the aperiodic and periodic spectra, respectively. The colored shaded areas along the line indicate the SE. Gray shaded areas indicate the analyzed frequency bands. Power is expressed in log10 (μV^2^) on the *y*-axis for normally spaced frequencies on the *x*-axis only for visualization purposes.

**Figure 2:**
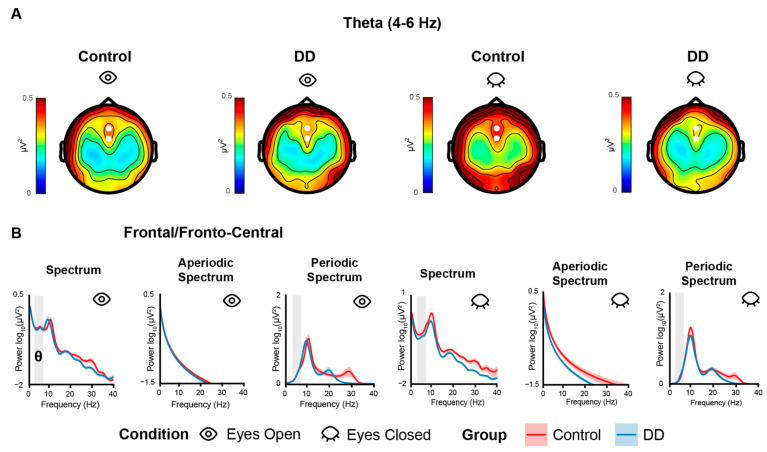
(**A**) Topographies of theta power calculated for each group (Control: 1st–3rd columns; DD: 2nd–4th column) and for each condition (EO: 1st–2nd column; EC: 3rd–4th). White dots indicate the channels used to compute theta power for statistical analyses. (**B**) Power spectra computed in Frontal/Fronto-Central electrodes for each group (Control: red line; DD: blue line) and condition (EO: 1st–3rd column; EC: 4th–6th column). Colored shaded areas along the line indicate the SE. Gray-shaded areas indicate the analyzed frequency bands. Power is expressed in log10 (μV^2^) on the *y*-axis for normally spaced frequencies on the *x*-axis only for visualization purposes.

Alpha (7–13 Hz) and beta (15–25 Hz) average power was extracted in the left (channels: PO7, PO3, O1) and right (channels: PO8, PO4, O2) parieto-occipital electrode cluster, while theta (4–6 Hz) was averaged across fronto-central channels Fz and FCz, considering the topographical distribution of power in each band (See Figure 1 and Figure 2). This was completed separately for the raw power spectrum and the periodic/FOOOF-corrected one. Offset and exponent values were averaged across the relevant electrode clusters for alpha, beta (parieto-occipital clusters) and theta (frontal/fronto-central channels). IAF values were extracted in the left and right parieto-occipital electrode clusters.

Repeated measures mixed-design analysis of variance (RM-Mixed ANOVA) was employed to analyze the mean alpha and beta power as well as IAF with condition (EC, EO) and cluster (Left, Right) as within factors and group (DD, Control) as a between factor with the ‘ez’ package in R statistics [59]. Theta power was analyzed in the same way but without cluster as a within factor.

The offset and exponent of the aperiodic component were analyzed in the same clusters of channels and with the same within-/between-subjects factors as for the alpha/beta or theta ANOVA. Considering that the focus of this work is aperiodic features of the power spectrum, the data of the 2 DD and 3 control participants were excluded from all statistical analyses as they showed offset and/or exponent values (averaged across left and right parieto-occipital clusters) outside ± 2 SD of their respective groups. Post hoc (i.e., planned comparisons) tests were run to explore possible interaction effects, and the relative *p*-values were first pooled separately for each effect and then FDR corrected [60].

Separate linear models were then implemented to study the possible relationship between reading tests scores (z-scores of words reading speed, pseudowords reading speed, text reading time, word reading error number, pseudowords reading error number, text reading error number) and the EEG measures extracted at resting-state averaged across conditions (EC, EO). Only the EEG measures which yielded significant group effects in the ANOVAs were used as possible linear predictors together with group (DD, Control). For each dependent variable, the best model was selected by retaining the model with the lowest Akaike Index (AIC) from all possible models via a forward- and backward-step selection process via the MASS package in R statistics [61]. Post hoc tests were run to explore possible interaction effects via the ‘emmeans’ package [62] in R. All *p*-values were FDR corrected. ANOVAs were run on each model to visualize the results more clearly (see Table 2). Additionally, Pearson’s correlation was used to test the relationship between offset and exponent independently from conditions, groups, and clusters. Results of statistical analyses are summarized in Section 3.

Additional exploratory correlations between EEG and behavioral measures are available in Supplementary Materials.

## 3. Results

### 3.1. EEG Results—Aperiodic Activity

The RM-mixed ANOVA on offset values extracted from parieto-occipital clusters showed a main effect of group (*F*_(1,49)_ = 7.06, *p* = 0.010, *η*2 = 0.099) revealing a lower offset for the DD group (*M* = 0.12, *SE* = 0.05) as compared to the Control group (*M* = 0.31, *SE* = 0.04). A main effect of condition was found (*F*_(1,49)_ = 10.57, *p* = 0.002, *η*2 = 0.03) showing a larger positive offset for the EC condition (*M* = 0.27, *SE* = 0.04) with respect to the EO condition (*M* = 0.17, *SE* = 0.03). No other effect reached significance (all *ps* > 0.09).

The RM-Mixed ANOVA on exponent values extracted from parieto-occipital clusters showed only a main effect of group (*F*_(1,49)_ = 5.53, *p* = 0.022, *η*2 = 0.07), revealing a lower exponent for the DD group (*M* = 1.05, *SE* = 0.05) as compared to the Control group (*M* = 1.21, *SE* = 0.03). No other effect reached significance (all *ps* > 0.12). The RM-Mixed ANOVA on offset values extracted from frontal electrode sites only showed an effect of Condition (*F*_(1,49)_ = 6.53, *p = 0*.013, *η*2 *=* 0.33) reflecting a higher offset in the EC condition (*M* = 0.37, *SE* = 0.04) with respect to the EO condition (*M* = 0.28, *SE* = 0.03). No other effect reached significance (all *ps* > 0.07). The RM-Mixed ANOVA on exponent values extracted from frontal electrode sites did not show significant effects (all *ps* > 0.69).

### 3.2. EEG Results—Periodic Activity

**Figure 3:**
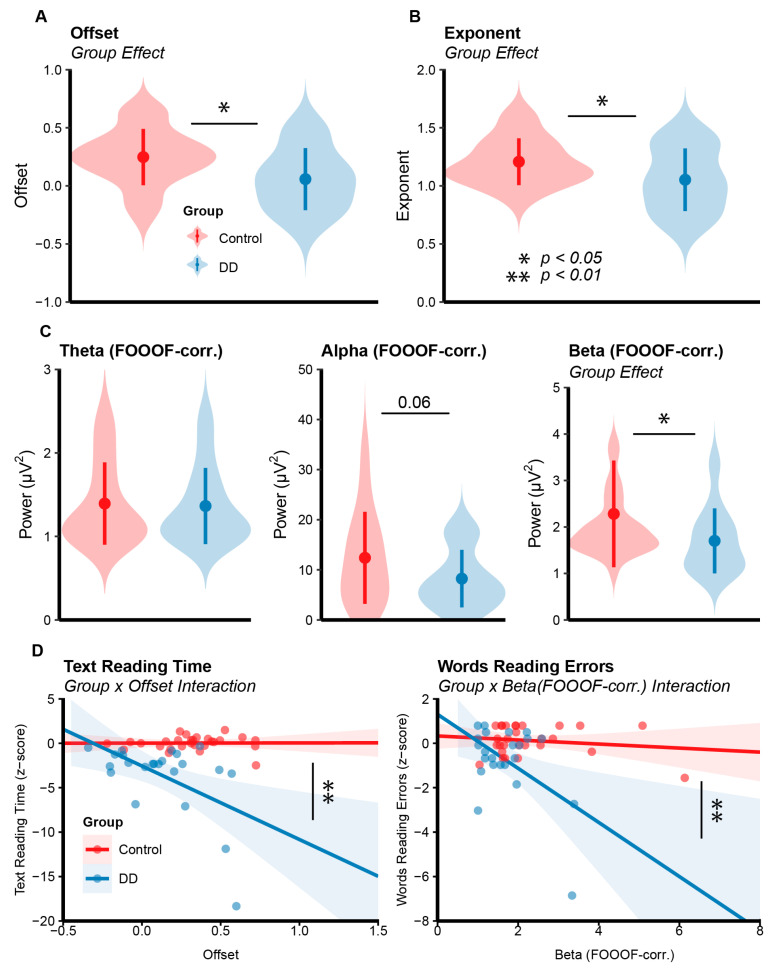
(**A**,**B**) Results of EEG data analysis. Group effects for Offset and Exponent parameters computed via FOOOF for each group (Control: red, DD: blue). Points represent the mean value for each group with SD bars. Violin plots depict data distributions. (**C**) Group averages for theta and alpha and group effects for beta power corrected via FOOOF for each group. (**D**) Interaction effects from the analysis of linear models for Text Reading Time (1st column) and Words Reading Time (2nd column). Shaded areas represent SE.

The RM-Mixed ANOVA on alpha power showed only a main effect of condition (*F*_(1,49)_ = 31.99, *p* < 0.001, *η*2 = 0.160), revealing a larger alpha power for the EC condition (*M* = 2.80, *SE* = 0.42) with respect to the EO condition (*M* = 0.72, *SE* = 0.18). A tendency to significance was found for the group effect (*F*_(1,49)_ = 3.23, *p* = 0.078), showing a tendency of a lower alpha power in the DD group (*M* = 1.23, *SE* = 0.28) with respect to the Control group (*M* = 2.19, *SE* = 0.43). No other effect reached significance (all *ps* > 0.10). The same analysis run on the alpha-corrected power (i.e., extracted from the periodic spectrum computed via FOOOF) also showed a main effect of condition (*F*_(1,49)_ = 66.92, *p* < 0.001, *η*2 = 0.25), revealing larger alpha power for the EC (*M* = 15.90, *SE* = 1.64) with respect to the EO (*M* = 5.11, *SE* = 0.84) condition. A tendency to significance was found for the group effect *(F*_(1,49)_ = 3.56, *p* = 0.064), showing a numerical tendency of a weaker alpha power in the DD group (*M* = 8.23, *SE* = 5.74) with respect to the Control group (*M* = 12.40, *SE* = 9.18). No other effect reached significance (all *ps* > 0.07) (Figure 3).

The RM-Mixed ANOVA on beta power showed an interaction effect between group and condition (*F*_(1,49)_ = 4.11, *p* = 0.047, *η*2 = 0.02). Post hoc t-tests showed a larger beta power for the EC (*M* = 0.23, *SE* = 0.03) with respect to the EO (*M* = 0.12, *SE* = 0.01) condition both for the Control group (*t*_(27)_ = −5.12, *p* < 0.001) and the DD group (*t*_(22)_ = −2.59, *p* = 0.041; EC: *M* = 0.15, *SE* = 0.02; EO: *M* = 0.09, *SE* = 0.01). The interaction may have been partially driven by a larger difference between conditions (EC vs. EO) found in the Control group (*M* = 0.11, *SE* = 0.02) with respect to the DD group (*M* = 0.05, *SE* = 0.02), but such a test only showed a tendency to significance (*t*_(48)_ = 2.06, *p* = 0.072). A main effect of condition was also found (*F*_(1,49)_ = 29.56, *p* < 0.001, *η*2 = 0.10), revealing larger beta power for the EC condition (*M* = 0.19, *SE* = 0.02) with respect to the EO condition (*M* = 0.10, *SE* = 0.01). No other effect reached significance (all *ps* > 0.12). The same analysis run on beta-corrected power showed a main effect of group (*F*_(1,49)_ = 4.50, *p* = 0.038, *η*2 = 0.07), revealing a larger beta power for the Control group (*M* = 2.28, *SE* = 0.21) with respect to the DD group (*M* = 1.70, *SE* = 0.14). A main effect of condition (*F*_(1,49)_ = 55.90, *p* < 0.001, *η*2 = 0.12) was also found revealing larger beta power for the EC condition (*M* = 2.42, *SE* = 0.17) with respect to the EO condition (*M* = 1.62, *SE* = 0.12) for both experimental groups (Figure 3).

The RM-Mixed ANOVAs on theta power and theta-corrected power did not show significant effects (all *ps* > 0.08). The RM-Mixed ANOVAs run on offset and exponent extracted from frontal/fronto-central electrodes (i.e., as for theta power) did not show significant effects (all *ps* > 0.25 and > 0.81, respectively) (Figure 3).

The RM-Mixed ANOVA run on IAF values revealed no significant effects (all *ps* > 0.30), while the same analysis run on IAF extracted from the periodic spectrum computed via FOOOF revealed a main effect of cluster (*F*_(1,49)_ = 5.03, *p* = 0.029, *η*2 = 0.01), showing a slightly higher IAF on the left parieto-occipital cluster (*M* = 10.60, *SE* = 0.14) with respect to the right one (*M* = 10.30, *SE* = 0.15).

### 3.3. Linear Models and Correlation Results

Only the EEG measures (i.e., offset, exponent, raw beta power, FOOOF-corrected beta power) which yielded significant effects in the ANOVAs were used as possible linear predictors of reading performance. The main effects of group in linear models are reported but not further commented, as they have been already addressed in the Participants section (see also Table 1).

The ANOVA on the linear model of words reading speed yielded a main effect of offset (*F*_(1,48)_ = 4.40, *p* = 0.04), showing that the larger the offset, the slower the reading speed (*M* = −1.26, *SE* = 0.60) irrespectively of the group. A main effect of group was also found (*F*_(1,48)_ = 37.63, *p* < 0.001). The ANOVA on the linear model of pseudowords reading speed showed a main effect of group (*F*_(1,49)_ = 36.37, *p* < 0.001) and no other main effects.

The ANOVA on the linear model of text reading time showed an interaction effect between group and offset (*F*_(1,47)_ = 9.03, *p* = 0.004). The post hoc test investigating the between-groups difference in the predictor slopes was significant (*t*_(47)_ = 3.00, *p* = 0.004), revealing a stronger negative relationship between text reading time and offset in the DD group (*M* = −8.27, *SE* = 1.95) with respect to the Control group (*M* = 0.02, *SE* = 1.94). Thus, the higher the offset, the longer the text reading time in DD participants; the same relationship was almost null in Control participants. Moreover, the main effects of group (*F*_(1,47)_ = 27.16, *p* < 0.001) and offset were found (*F*_(1,47)_ = 8.85, *p* = 0.004); this latter result was not further analyzed considering the interaction effect between group and offset.

The ANOVA on the linear model of word reading errors revealed an interaction effect between group and beta power (FOOOF-corr.) (*F*_(1,47)_ = 8.66, *p* = 0.005). A post hoc test investigating the between-groups difference in predictor slopes was significant (*t*_(47)_ = 2.94, *p* = 0.005), revealing a stronger negative relationship between word reading errors and beta power (FOOOF-corr.) in the DD group (*M* = −1.21, *SE* = 0.33) with respect to the Control group (*M* = −0.09, *SE* = 0.18). Thus, the higher the beta power (FOOOF-corr.), the higher the number of errors in DD participants, while this relationship was almost null in Control participants. A main effect of group was also found (*F*_(1,47)_ = 8.23, *p* < 0.006). A main effect of beta (FOOOF-corr.) was found (*F*_(1,47)_ = 4.75, *p* = 0.03), but it was not further discussed considering its involvement in the interaction between group and beta power (FOOOF-corr.).

The ANOVAs on the linear model on pseudowords and text reading errors only yielded a main effect of group (*F*_(1,49)_ = 10.24, *p* = 0.002 and *F*_(1,49)_ = 24.18, *p* < 0.001, respectively).

A positive correlation between offset and exponent values averaged across conditions (EC, EO), clusters (left parieto-occipital, right parieto-occipital) and groups (Control, DD) was found (*ρ*_(49)_ = 0.79, *p* < 0.001), showing that higher offsets were associated with a higher exponent.

### 3.4. Results Overview

Overall, the results showed significant alterations of aperiodic and periodic activity in participants with DD. In particular, lower offset and exponent values as well as lower beta power (FOOOF-corrected) were found in the DD group with respect to the Control group. Importantly, such alterations were found to be predictive of reading skills but only in the DD group. In particular, higher offset values and higher beta power (FOOOF-corrected) were associated to worse performance in text and word reading tasks, respectively. These results provide additional evidence about alterations in neural noise and/or cortical excitability as well as magnocellular impairments in adults with Developmental Dyslexia. If such alterations also apply to children, their potential use as markers of Developmental Dyslexia might foster its early diagnosis.

## 4. Discussion

The main aim of this study was to clarify the distinct role of periodic and aperiodic endogenous neural activity in DD and test their potential as neurophysiological markers that could contribute to the improvement of the DD diagnostic process and to a better understanding of interindividual variability in reading. To achieve this goal, we analyzed resting-state EEG from adults with DD and typical readers, testing for possible differences regarding aperiodic-corrected and uncorrected oscillatory indexes (i.e., theta, alpha, and beta power and IAF) and aperiodic ones (i.e., exponent and offset). We also measured the possible modulations of these indexes between eyes-closed and eyes-open conditions as well as their role in predicting reading performance on standardized neuropsychological tests. Our results reveal that DD participants showed significant alterations of the aperiodic indexes and a significantly decreased aperiodic-adjusted power in the beta band compared to typical readers. Moreover, in the DD group, aperiodic offset and beta power emerged as significant predictors of reading skills in text and words reading tasks, respectively.

In the DD group, we observed a significantly decreased exponent at parieto-occipital sites, reflecting a flatter power spectrum [28]. On the physiological level, exponent changes are thought to reflect alterations in neuronal excitability/inhibition balance, with a flattened spectrum describing a preponderance of excitation over inhibition [33,34]. Within the dynamic network communication framework, this state of increased excitability has been related to a reduced precision of temporal coupling between specific phases of oscillatory cycles and neuronal spiking, introducing a higher level of noise with possible consequences for neural communication efficiency [35]. In addition, it has been suggested that the extension of this model could explain behavioral and neurological symptoms of different psychiatric and neurological conditions [35]. As regards the DD population, previous evidence pointed to possible increased neuronal excitability arising from a genetic-related alteration in glutamatergic signaling and neuronal migration within key areas for reading processes [49]. The concomitant neural noise increase has been proposed to have a role in across-domains reading-related impairments shown by individuals with DD, from low-level auditory processing and perceptual noise exclusion to phonological awareness and phoneme–grapheme multisensory integration [49,63]. Therefore, considering our results, the aperiodic exponent may represent a novel and easily measurable index of neural noise in DD, which could in turn be linked to its pathophysiology and to the interindividual variability in reading-related difficulties. Alterations of the aperiodic exponent have been described in other neurological and psychiatric conditions, enhancing the interest in studying its potential role as a putative biomarker in clinical populations. However, the available evidence is limited; thus, inferences about possible shared mechanisms underlying different diseases are still premature [4].

The aperiodic component of the power spectrum is also characterized by its offset. As seen in previous studies [39,40], here, we also observed a positive correlation between offset and exponent regardless of the group. Another general, group-independent observation was that a higher offset emerged in the EC as compared to the EO condition, which is a difference that could be attributed to the higher level of inhibition characterizing the EC condition as consistent with a previous report [39]. These two results, overall, suggest that not only the exponent but also the offset seem connected to a modulation of E/I ratio. Importantly, in the present study, the offset was significantly reduced at parieto-occipital channels for the DD group compared to control participants. Such an alteration describes a relative downward broadband shift of power that has been linked to variations of the firing rate in specific neural populations [36]. Furthermore, broadband changes in the ECoG power spectrum were shown to dynamically mark local neural activity across different visual, linguistic, and motor tasks [64], underlining its potential relevance in relation to cognitive processes. Moreover, particularly for DD participants, we observed a link between the aperiodic offset and text reading time, indicating that a slower performance corresponds to higher offset values. Considering the observed strong positive correlation between exponent and offset across groups, although distinct physiological mechanisms underlie these indexes, increasing offset values seem to correspond to increasing exponent values, indicating lower neural noise levels. However, upward broadband shifts have also been linked to increased neuronal firing rates in previous studies [36]; thus, whether a relationship between such shifts of the spectrum and variations in endogenous noise levels exists remains currently unknown, pointing toward the necessity of further investigations.

These results may represent an important step forward in understanding the complex relationship between reading performance and indexes of neural noise in DD. The neural noise hypothesis of DD suggests that a higher level of hyperexcitability (i.e., neural noise) would hamper neural synchronization, which is an important feature for virtually any neural computation, from basic sensory processing [65] to multisensory integration [66] and speech perception [67,68]. For example, the ability to encode speech is thought to be grounded on the synchronization between neural oscillations and periodic features of speech sounds [67] and on the integration of both fast (e.g., phonemic) and slow (e.g., prosodic) rhythms to achieve meaningful linguistic representations [68]. The noise-related impairment of neural synchronization could prevent individuals with DD from acquiring robust phonemic representations, thus determining low phonological awareness and consequently impaired phoneme–grapheme mapping, which is essential for reading [69]. Moreover, higher levels of internal noise would prevent individuals with DD from successfully excluding external noise during speech perception [70]. Finally, it could impact perceptual noise exclusion in the visual domain, for example the ability to efficiently extract letters/words in crowded texts [71].

We also found that individuals with DD showed weaker resting-state beta-band power with respect to control participants. In addition, we report a relationship between resting-state beta (FOOOF-corrected) power and the number of errors committed during reading tests: the higher the beta power, the higher the number of errors (i.e., more negative z-scores; see Figure 3) in DD participants, while this relationship appeared as being almost null in control participants. Beta-band oscillations (15–25 Hz) are thought to represent the “natural rhythms” of parietal cortices [72,73]. More specifically, beta band activity in parietal and fronto-parietal sites was repeatedly associated with a plethora of visual processes such as visual detection [74] and identification [75], visual search [7], motion discrimination [76] and spatial attention [77]. Importantly, the modulation of beta power in parietal areas has been robustly associated with the visual discrimination of letters in crowded displays [78,79,80], assuming a key role for the neurophysiological characterization of reading processes and consequently for the study of reading impairments. Relatedly, individuals with DD suffer from excessive visual crowding during reading [71,81,82], which is a phenomenon that impacts magnocellular-dorsal (M-D) stream functionality in the brain [83] considering its role in the contour integration/segregation of visual objects. In this regard, different studies showed impairment of different functions linked to the M-D stream in individuals with DD [84,85,86,87] such as visual search [88] and visuo-spatial attention [89,90] leading to the development of the magnocellular-dorsal theory of DD. The alteration of resting-state beta power and its relationship with reading accuracy might provide another potential clue about the M-D-related deficits in individuals with DD considering the association between parietal beta oscillations and M-D functionality, given that parietal areas are the main projection of the M-D stream. Further studies could investigate the relationship between resting-state and task-related beta power during reading and/or other cognitive tasks to deepen the understanding of the specific functional role of beta oscillations and its precise link with the M-D stream functionality.

Lastly, our data showed a tendency toward significance of a decrease in alpha power for the DD group compared to controls both for raw and aperiodic-adjusted power. Although this result appears to be in line with some previous findings of significant alterations of endogenous alpha rhythms in DD individuals as opposed to typical readers [8], this evidence is not consistently replicated in the literature. Furthermore, consistently with previous studies [15,91], there was no significant between-group difference when investigating endogenous individual alpha frequency (IAF) in neither the raw nor the FOOOF-corrected spectrum.

While we show here that the FOOOF algorithm can provide relevant information to characterize the pathophysiological mechanisms in DD, the interpretation of the underlying physiological alterations is still a matter of investigation, as discussed above. In particular, given the limited amount of studies investigating the relationship between aperiodic measures and tasks probing specific cognitive domains, the functional specificity of such parameters with respect to the neuropsychological profile of DD still needs to be established. Moreover, the present study focused the investigation on aperiodic activity (and its impact on periodic activity) during the resting state; therefore, further studies are needed to clarify if similar alterations would apply to task-related activity.

A limitation of the present study is the relatively small sample size (DD = 26, C = 31). This limitation is common to previous studies investigating endogenous oscillatory activity in DD, which have a comparable sample size (mean DD = 22.14, SD = 5.37, range = 14–29) [10,13,14,15,16,17,18]. A recent systematic review on EEG studies in children with DD underlined this aspect as a common issue potentially affecting also task-related investigations [8]. Future works need to address the results’ replicability in broader samples, overcoming the limited recruiting capacity of a single laboratory possibly through multicenter collaborative studies.

## 5. Conclusions

The present study provides the first evidence about alterations of aperiodic EEG activity in DD as well as preliminary evidence about its relationship with reading performance. The application of the FOOOF algorithm allowed us to isolate pure oscillations from aperiodic activity, highlighting alterations of parieto-occipital beta-band activity in DD and their potential link with reading performance. The use of this methodological innovation might ameliorate the study of neural oscillations in future research while avoiding confounds driven by aperiodic activity in the interpretation of results.

Our results support the presence of an increase in neural noise levels, which could impair a number of different sensory and cognitive domains. In particular, considering that higher levels of neural noise possibly index suboptimal neural spike timing, they might impact the functional connectivity of key hubs for reading and several other cognitive processes in DD by disrupting cortical synchronization. Therefore, future studies on functional connectivity in DD might benefit from taking into consideration the impact of neural noise from a theoretical standpoint as well as considering how it might affect connectivity measurements.

Considering the complex and multifaceted nature of DD [92], a comprehensive analysis of periodic and aperiodic neural indexes holds the potential to deepen and integrate different theoretical considerations, going beyond a mutual-exclusion perspective. The observed group-specific relations between endogenous neural dynamics and reading abilities may have important translational implications for clinical contexts, since the use of objective and easily measurable neurophysiological indexes could greatly improve standard neuropsychological assessment.

## Figures and Tables

**Table 1:** Descriptive statistics of Control and DD participants for each cognitive test and independent sample *t*-tests’ results. All measures are reported in z-scores except for Raven’s APM.

Measure	Controls (*n* = 28) Mean (SD)	DD (*n* = 23) Mean (SD)	*p*-Value
Raven’s APM	7.96 (2.26)	7.13 (2.80)	0.256
Words Reading Speed	0.79 (1.15)	−1.05 (1.05)	<0.001
Pseudowords Reading Speed	0.82 (1.47)	−1.30 (0.91)	<0.001
Text Reading Time	0.02 (0.77)	−3.57 (4.12)	<0.001
Words Reading Errors	0.12 (0.65)	−0.76 (1.67)	0.023
Pseudowords Reading Errors	0.65 (0.54)	−0.28 (1.40)	0.007
Text Reading Errors	0.75 (0.60)	−0.75 (1.48)	<0.001

**Table 2:** Results of the ANOVAs performed on linear models.

**Words Reading Speed**			**Words Reading Errors**		
	**F** **(df)**	***p***		**F** **(df)**	***p***
Group	37.63 (1, 48)	<0.001	Group	8.23 (1, 47)	0.006
Offset	4.40 (1, 48)	0.041	Beta (FOOOF-corr.)	4.75 (1, 47)	0.034
			Group x Beta (FOOOF-corr.)	8.66 (1, 47)	0.005
**Pseudowords Reading Speed**			**Pseudowords Reading Errors**		
	**F** **(df)**	***p***		**F** **(df)**	***p***
Group	36.37 (1, 49)	<0.001	Group	10.24 (1, 49)	0.002
**Text Reading Time (Syll/Sec)**			**Text Reading Errors**		
	**F** **(df)**	***p***		**F** **(df)**	***p***
Group	27.16 (1, 47)	<0.001	Group	24.18 (1, 49)	<0.001
Offset	8.85 (1, 47)	0.004			
Group x Offset	9.03 (1, 47)	0.004			

x represents an interaction effect.

## Data Availability

Data are available upon requests to the Authors.

## Supplementary Materials

The following supporting information can be downloaded at: https://www.mdpi.com/article/10.3390/biomedicines11061607/s1, Figure S1: Correlations (Parietal clusters)–Eyes closed condition; Figure S2: Correlations (Parietal clusters)–Eyes open condition; Figure S3: Correlations (Fronto-central cluster).
