# Supplementary material for: Periodic and Aperiodic EEG Features as Potential Markers of Developmental Dyslexia
Source: Biomedicines. 2023 Jun 1;11(6):1607. doi: 10.3390/biomedicines11061607 (PMC10296084; doi:10.3390/biomedicines11061607)
Supplement: Supplementary file 1 [file biomedicines-11-01607-s001.zip › biomedicines-2346308-supplementary.pdf]

Supplementary Materials

Correlations (Parietal clusters) – Eyes closed condition

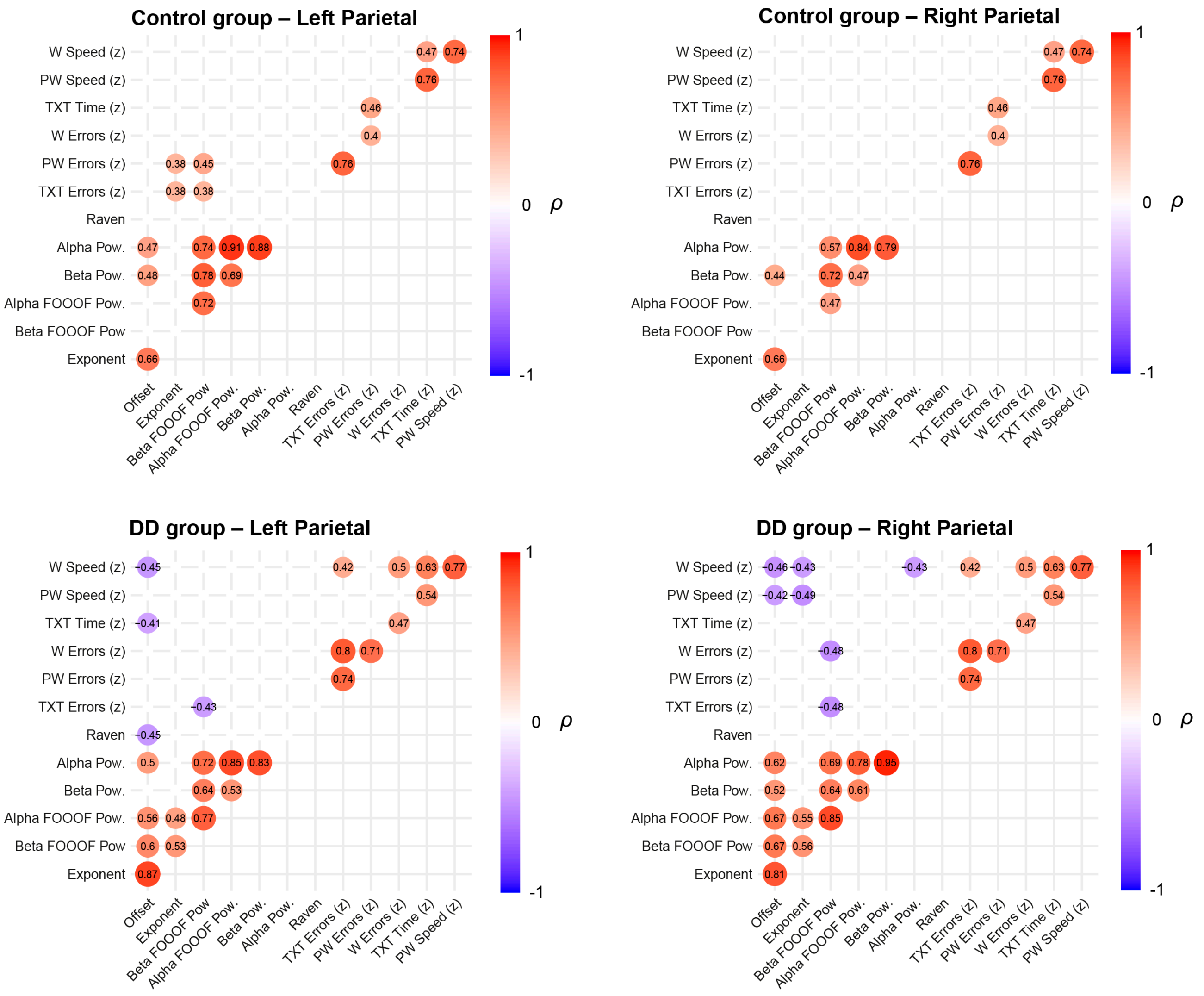

Figure S1. Correlations of EEG variables recorded in the eyes closed condition for each group (Control: 1st row, DD 2nd row) and each parietal cluster (Left cluster: 1st column, Right parietal: 2nd column) and behavioral variables. Behavioral values (W/PW Speed/Errors: Word/Pseudowords reading speed/errors, TXT Time/Errors: Text reading time/errors) are expressed in z-scores except for Raven (Raven's APC) which is expressed in its raw score. Numbers embedded within colored circles represent Pearson's ρ for each correlation. Circles's radius grows in function of ρ magnitude (absolute). Colors indicate positive (red) or negative (blue) correlations). Only correlation with a p-value (FDR corrected) < 0.05 are shown.

Correlations (Parietal clusters) – Eyes Open condition

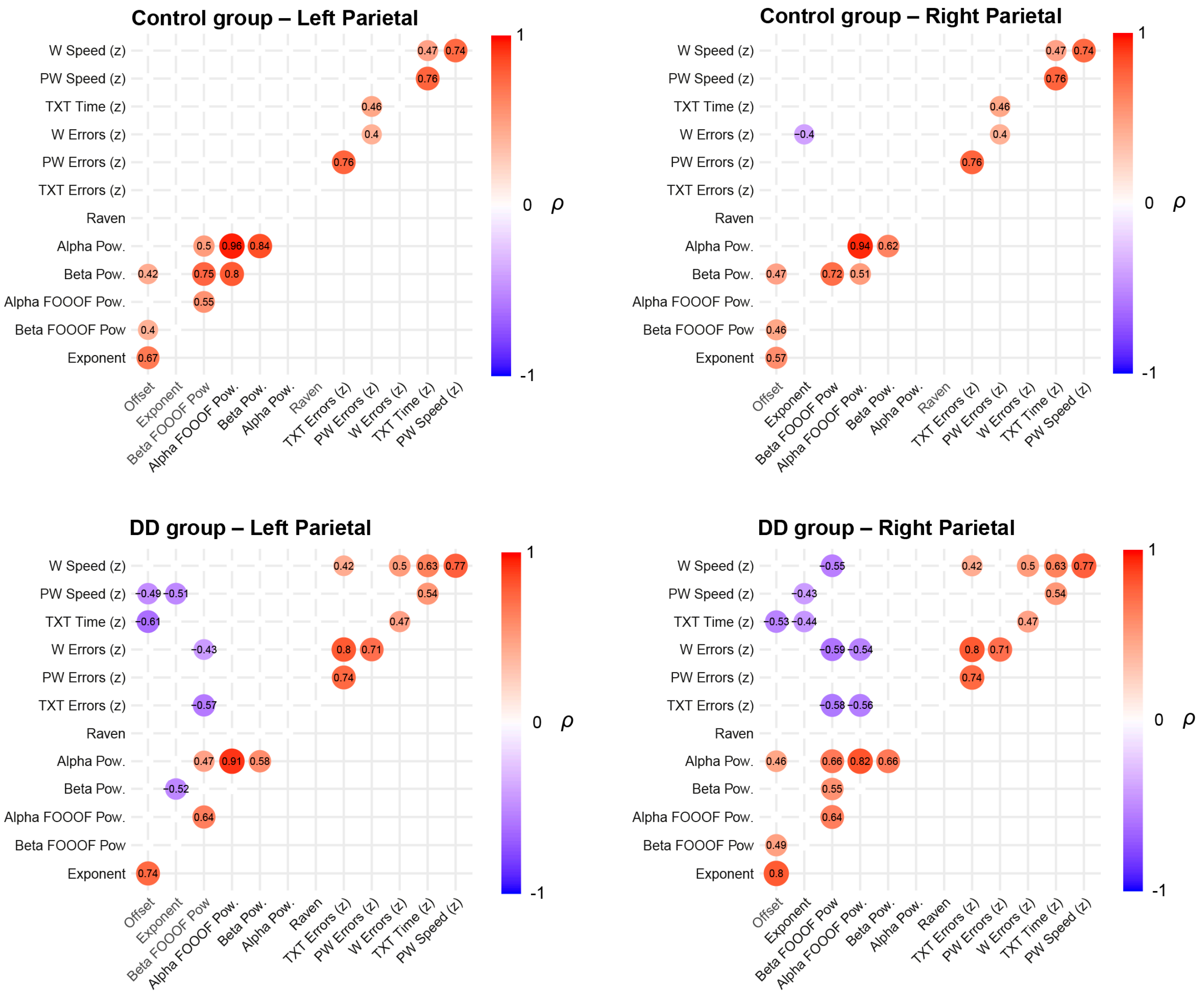

Figure S2. Correlations of EEG variables recorded in the eyes open condition for each group (Control: 1st row, DD 2nd row) and each parietal cluster (Left cluster: 1st column, Right parietal: 2nd column) and behavioral variables). Behavioral values (W/PW Speed/Errors: Word/Pseudowords reading speed/errors, TXT Time/Errors: Text reading time/errors) are expressed in z-scores except for Raven (Raven's APC) which is expressed in its raw score. Numbers embedded within colored circles represent Pearson's ρ for each correlation. Circles's radius grows in function of ρ magnitude (absolute). Colors indicate positive (red) or negative (blue) correlations). Only correlation with a p-value (FDR corrected) < 0.05 are shown.

Correlations (Fronto-central cluster)

Eyes Closed condition

Eyes Open condition

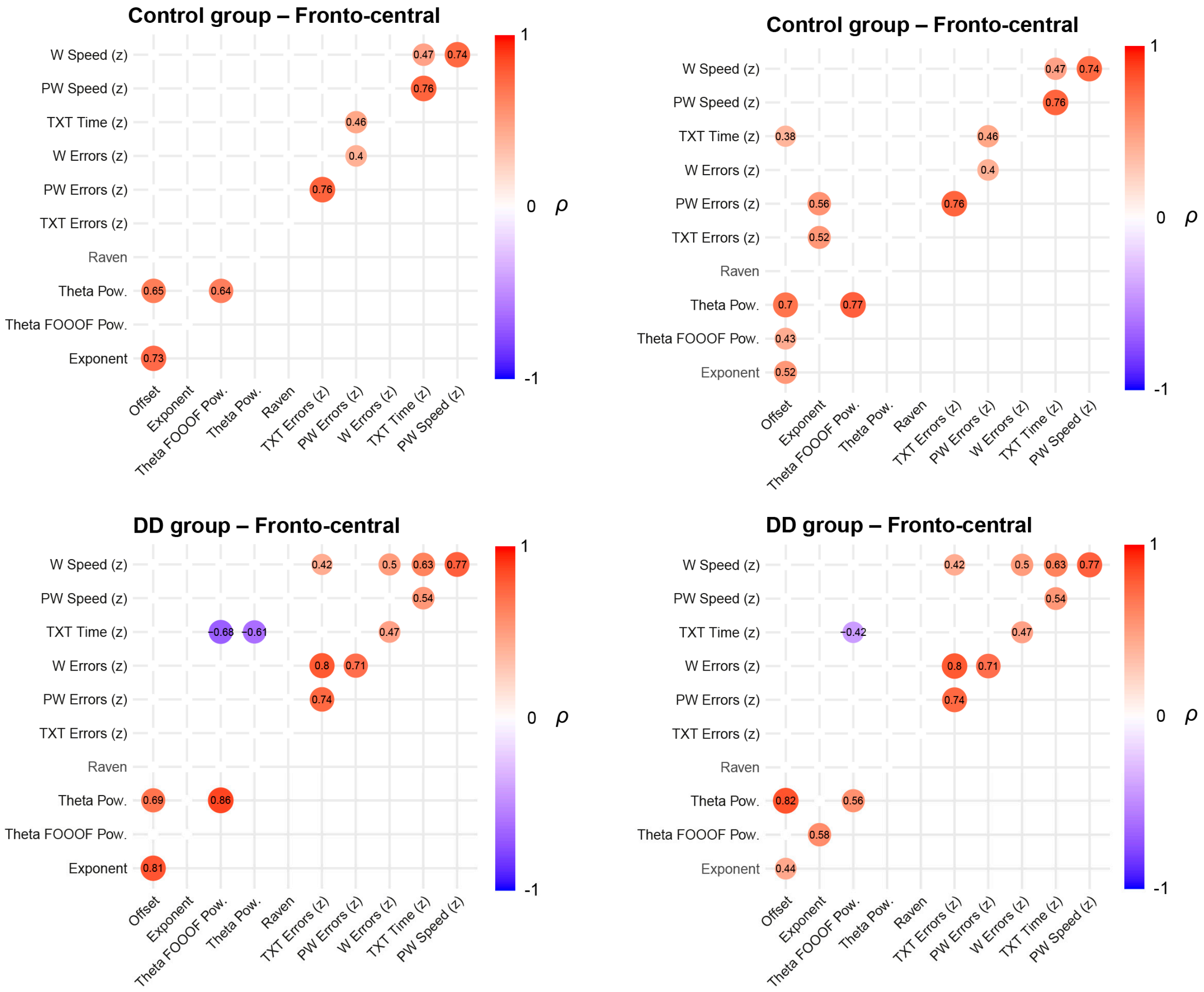

Figure S3 Correlations of EEG variables recorded from fronto-central cluster for each condition (Eyes closed: 1st column, Eyes open: 2nd column) and each group (Control: 1st row, DD 2nd row). Behavioral values (W/PW Speed/Errors: Word/Pseudowords reading speed/errors, TXT Time/Errors: Text reading time/errors) are expressed in z-scores except for Raven (Raven's APC) which is expressed in its raw score. Numbers embedded within coloured circles represent Pearson's ρ for each correlation. Circles's radius grows in function of ρ magnitude (absolute). Colors indicate positive (red) or negative (blue) correlations). Only correlation with a p-value (FDR corrected) < 0.05 are shown.
